# The Pension Fund for Nurses

**Published:** 1887-07-09

**Authors:** M. Clement

**Affiliations:** Lady Superintendent, Association of Nurses. Western Road, Brighton


### THE PENSION FUND FOR NURSES.

I should be glad to obtain some particulars as to your Pension Fund for Nurses ; also cards to be hung up in the nurses' sitting-rooms. I am the Superintendent of different Nursing Homes, as the Tunbridge Wells Nursing Institution ; also in Worthing, and one in North London (Highbury). The homes are now all well established, and self-supporting. I have a staff of sixty to eighty nurses, some paid by salary, whilst some work on the commission principle. No doubt many of them would like to enter their names if I approve of the scheme.—Yours faithfully,

M. Clement,
Lady Superintendent, Association of Nurses.
Western Road, Brighton.

